# New Books

**Published:** 2010-10

**Assessing the Effects of the Gulf of Mexico Oil Spill on Human Health**

Margaret A. McCoy, Judith A. Salerno, eds.

Washington, DC:National Academies Press, 2010. 200 pp. ISBN: 978-0-309-15781-0, $33

**Bioethics in Singapore: The Ethical Microcosm**

John M. Elliott, W. Calvin Ho, Sylvia S.N. Lim, eds.

Hackensack, NJ:World Scientific, 2010. 344 pp. ISBN: 978-981-4327-10-7, $78

**Blowout in the Gulf: The BP Oil Spill Disaster and the Future of Energy in America**

William Freudenburg, Robert Gramling

Cambridge, MA:MIT Press, 2010. 240 pp. ISBN: 978-0-262-01583-7, $18.95

**Changing Climates, Earth Systems and Society**

John Dodson, ed.

New York:Springer, 2010. 360 pp. ISBN: 978-90-481-8715-7, $129

**Developing Adaptation Policy and Practice in Europe: Multi-level Governance of Climate Change**

E. Carina H. Keskitalo, ed.

New York:Springer, 2010. 376 pp. ISBN: 978-90-481-9324-0, $179

**Energy, Environment and Sustainable Development**

Mohammed Aslam Uqaili, Harijan Khanji

New York:Springer, 2010. 250 pp. ISBN: 978-3-7091-0108-7, $129

**Environmental Modeling and Health Risk Analysis (Acts/Risk)**

Mustafa M. Aral

New York:Springer, 2010. 462 pp. ISBN: 978-90-481-8607-5, $89.95

**Global Commons, Domestic Decisions: The Comparative Politics of Climate Change**

Kathryn Harrison, Lisa McIntosh Sundstrom, eds.

Cambridge, MA:MIT Press, 2010. 320 pp. ISBN: 978-0-262-51431-6, $25

**Global Governance of Hazardous Chemicals: Challenges of Multilevel Management**

Henrik Selin

Cambridge, MA:MIT Press, 2010. 240 pp. ISBN: 978-0-262-51390-6, $22

**Global Perspectives on Childhood Obesity: Current Status, Consequences and Prevention**

Debasis Bagchi, ed.

St. Louis, MO:Elsevier, 2011. 536 pp. ISBN: 978-0-12-374995-6, $149.95

**Impact of Climate Change on Natural Resource Management**

Bipal K. Jana, Mrinmoy Majumder, eds.

New York:Springer, 2010. 415 pp. ISBN: 978-90-481-3580-6, $169

**Increasing Access to Health Workers in Remote and Rural Areas through Improved Retention**

World Health Organization

Geneva:WHO Press, 2010. 76 pp. ISBN: 978-9-2415-6401-4, $25

**Knowledge and Environmental Policy: Re-Imagining the Boundaries of Science and Politics**

William Ascher, Toddi Steelman, Robert Healy

Cambridge, MA:MIT Press, 2010. 280 pp. ISBN: 978-0-262-51437-8, $23

**Regulating Chemical Risks: European and Global Challenges**

Johan Eriksson, Michael Gilek, Christina Rudén, eds.

New York:Springer, 2010. 350 pp. ISBN: 978-90-481-9427-8, $189

**Reviews of Environmental Contamination and Toxicology, Vol 209**

David M. Whitacre, ed.

New York:Springer, 2010. 110 pp. ISBN: 978-1-4419-6882-1, $129

**Sacrifice Zones: The Front Lines of Toxic Chemical Exposure in the United States**

Steve Lerner

Cambridge, MA:MIT Press, 2010. 368 pp. ISBN: 978-0-262-01440-3, $29.95

